# Effect of Bilateral Opercular Syndrome on Speech Perception

**DOI:** 10.1162/nol_a_00037

**Published:** 2021-07-13

**Authors:** Grant M. Walker, Patrick Sarahan Rollo, Nitin Tandon, Gregory Hickok

**Affiliations:** Department of Cognitive Sciences, University of California, Irvine; Department of Neurosurgery, University of Texas Medical School at Houston; Department of Language Science, University of California, Irvine

**Keywords:** speech perception, sensorimotor integration, audiovisual integration, bilateral surgical resection, opercular syndrome

## Abstract

Speech perception ability and structural neuroimaging were investigated in two cases of bilateral opercular syndrome. Due to bilateral ablation of the motor control center for the lower face and surrounds, these rare cases provide an opportunity to evaluate the necessity of cortical motor representations for speech perception, a cornerstone of some neurocomputational theories of language processing. Speech perception, including audiovisual integration (i.e., the McGurk effect), was mostly unaffected in these cases, although verbal short-term memory impairment hindered performance on several tasks that are traditionally used to evaluate speech perception. The results suggest that the role of the cortical motor system in speech perception is context-dependent and supplementary, not inherent or necessary.

## INTRODUCTION

Despite centuries of discussion and debate, the role of the motor system in speech perception continues to inspire curiosity and empirical investigations. More recently, the discovery of mirror neurons has renewed interest in motor theories of speech perception. These theories posit that motoric gesture representations play either a primary or supportive role in perceiving speech sounds via articulatory recoding (motoric simulation), thereby assisting the resolution of the indeterminacy problem in mapping from acoustic to phonological perception. Consistent with this theory, functional imaging research has shown conclusively that frontal motor speech-related areas activate during speech listening (Hickok et al., 2003; Watkins & Paus, 2004; Wilson et al., 2004) and transcranial magnetic stimulation (TMS) of premotor and primary motor areas can modulate performance on some perception tasks in an effector-specific manner (D’Ausilio et al., 2009; Möttönen & Watkins, 2012; Pulvermüller & Fadiga, 2010). For in depth reviews, discussion, and debate see Galantucci et al. (2006), Gallese et al. (2011), Hickok (2014), Hickok et al. (2011), Liebenthal and Möttönen (2018), Massaro and Chen (2008), Skipper et al. (2017).

Nowadays, researchers generally agree that a strong version of the motor theory—that motor speech systems are necessary for speech perception—is untenable. This conclusion is based on several facts. First, functional imaging evidence, where there is robust evidence for motor activation during perception, is, nonetheless, only correlational and cannot speak to the causal involvement of motor areas in perception. Second, while TMS has provided evidence of causation, these effects are typically small and only evident under noisy listening conditions or during tasks requiring explicit attention to sublexical phonological features (D’Ausilio et al., 2012; Sato et al., 2009), which are known to doubly dissociate from phonological ability under more ecologically valid conditions (Hickok & Poeppel, 2000, 2004, 2007). Third, evidence from the speech perception abilities of pre-lingual infants (Eimas et al., 1971), people with anarthria due to cerebral palsy (Bishop et al., 1990), and non-human animals (Kuhl & Miller, 1975) shows that a functioning motor speech system is not necessary for speech perception. Fourth, unilateral damage to motor speech areas, which can substantially disrupt speech fluency, does not produce a substantial (or in some cases any) concomitant deficit in speech perception (Rogalsky et al., 2011). Finally, acute unilateral deactivation of the left hemisphere causing complete expressive mutism during Wada procedures leaves word comprehension relatively spared (Hickok et al., 2008).

In light of such observations, most researchers promote a more nuanced view of the role of the motor system in receptive speech, arguing for a *modulatory* role that may engage, for example, under noisy listening conditions (Hickok et al., 2011; Skipper et al., 2017; Wilson, 2009). Accordingly, the research questions are becoming more nuanced as well, with an aim toward quantifying the magnitude of motor influence and specifying the condition(s) under which it holds (Skipper et al., 2017). For example, Stokes et al. (2019) used a behavioral psychometric approach to estimate the effect size of motor interference (articulatory suppression) on minimal pair perception in noise. They reported that motor interference reduced speech perception under noisy conditions by an average of approximately 1 dB, which is to say that increasing the stimulus volume by just 1 dB was enough to overcome the perceptual decrement of motor suppression. In two studies, another group compared the effects of TMS stimulation under two different task conditions, a sublexical task (4-alternative forced-choice syllable identification) (D’Ausilio et al., 2009) and a word comprehension task (Schomers et al., 2015). (Recall that these tasks are known to dissociate, as mentioned above.) Motor stimulation significantly increased errors only for the sublexical task, resulting in between 5–10% change in accuracy under noisy conditions, which approximates a 1 dB size effect (Stokes et al., 2019). The effects of motor stimulation during word comprehension affected only reaction times but not accuracy. Thus, existing evidence suggests that motor modulation during speech perception has an effect size of approximately 1 dB, holding only under noisy listening conditions, and may be less during normal comprehension compared to artificial laboratory tasks.

The present study contributes to this body of evidence via a detailed study of two cases of opercular syndrome. Clinical symptoms of opercular syndrome include severe disruption of voluntary control of the orofacial, lingual, pharyngeal, and masticatory muscles. Speech articulation is severely impaired, often to the point of mutism, despite automatic control of this musculature being relatively spared (Desai et al., 2013; Groswasser et al., 1988; Milanlioglu et al., 2013; Silveri et al., 2017). The syndrome is typically associated with bilateral lesions of the frontal operculum, including speech-related zones, although unilateral and subcortical lesions have also been documented (Bakar et al., 1998; Starkstein et al., 1988). Various etiologies, such as epileptic disorders, cerebrovascular events, degenerative diseases, or CNS infections, can cause opercular syndrome, which can be acquired or congenital, and persistent or intermittent. The syndrome is also known as Foix-Chavany-Marie syndrome, and the developmental form in children is known as Worcester-Drought syndrome (Christen et al., 2007). Bilateral perisylvian polymicrogyria is a developmental condition that also can lead to some of the symptoms observed in opercular syndrome, often severely affecting speech production and speech perception together, but without severely compromising other cognitive abilities (i.e., IQ); however, polymicrogyria is generally not specific to motor systems and often affects sensory cortices in the perisylvian region as well (Boscariol et al., 2010; Jansen et al., 2005; Saletti et al., 2007). Opercular syndrome due to surgical resection, as we report here, offers a unique opportunity to study the effects of severe and specific motor speech disruption due to bilateral cortical/subcortical lesions on receptive speech ability.

Two patients who had been clinically diagnosed with opercular syndrome based on clinical examination, medical reports, and neuroimaging were referred to us for an in-depth assessment by the neurosurgeon managing their current care. During multiple visits over the course of several months, the patients completed informal interviews and a comprehensive examination of their receptive speech abilities, as well as undergoing new, structural neuroimaging with MRI to provide a precise delineation of the surgical lesions’ anatomical extent. In our discussion of these cases, we consider how the specific patterns of performance and anatomical disruption bear on the limits of the motor system’s contributions to speech perception at the phonemic, word, and sentence levels, as well as to audio-visual integration of speech signals.

## MATERIALS AND METHODS

### Participants

Two patients with opercular syndrome due to bilateral surgical resection were tested on speech perception and language comprehension tasks. A control sample, selected for convenience, included four neurologically healthy lab members (3 male, 1 female), ranging 24–26 years in age, who performed a subset of the same tasks under identical testing conditions as the patients. Not all tasks were performed, as some of them were only administered to one of the patients during post-hoc, follow-up investigation of observed behavioral effects. All study procedures were performed after approval from the UT Health Committee for the Protection of Human Subjects. All subjects provided written informed consent. For details about the patients’ motor and communicative abilities at time of testing, see Additional Observations in the Results section.

#### Case history 1

Patient is a 27-year-old female with a history of seizures that began at age 15. The patient's intractable epilepsy was managed by a pediatric neurosurgeon (not the author, N. T.) at the age of 16. Prior to surgery, the Weschler Abbreviated Scale of Intelligence (WASI) indicated average Full Scale IQ (100), average Verbal IQ (93), and average Performance IQ (106), with noted weaknesses in verbal fluency to semantic cueing, naming to confrontation, working memory, sustained attention, and speeded sequential processing. She underwent invasive seizure monitoring with subdural grid electrodes and then several resective operations. First, a resection of right frontal opercular epileptogenic focus and, 6 months later, an anterior 2/3 corpus callosotomy. The patient then had an additional subdural grid implantation on the left side, followed by a left frontal topectomy and left parietal sub-pial transections. Two months after this last resective operation, the patient had a ventriculoperitoneal shunt placed and, at age 18, had a vagus nerve stimulation device implanted. The patient continues to suffer from seizure disorder. No postoperative neuropsychological testing was done. At the time of enrolling in the current study, she had not spoken in 9 years, since her last surgery.

#### Case history 2

Patient is a 54-year-old female with a history of focal seizures that began when she was 12. At the age of 18 she suffered a stroke secondary to the bleeding from a cavernous malformation of the left side of the brain. Multiple other cavernous hemangiomas were identified in the brain at age 22. The patient had a left frontal opercular cavernoma resected about 20 years ago. This led to a marked impairment of her language function and then a recovery over many months. She then had a hemorrhagic stroke in the right frontal lobe. A second resection was performed on the left side at age 42. She has had a total of nine spontaneous episodes of hemorrhagic strokes. The patient continues to suffer from seizure disorder, with fewer seizures and less intense seizures than when she was younger. She has a spastic hemiparesis on her left side. Preoperative neuropsychological exam results: On the WASI, she obtained a Full Scale IQ of 90. Her Performance IQ (99) was much higher than her Verbal IQ (86). Achievement screening with the Wide Range Achievement Test–3rd edition (Snelbaker et al., 2001) indicated average performance on a reading subtest (Standard Score: 107, post-HS grade equivalent) and a spelling subtest (Standard Score: 92, HS grade equivalent), and low average performance on a math subtest (Standard Score: 83, sixth grade equivalent). Neuropsychological testing indicated relative reductions in verbal fluency, fine motor speed and coordination, recall of rote verbal and nonverbal information following a delay, mental flexibility, and problem solving. She also reported moderate anxiety and moderate-to-severe depression. No postoperative neuropsychological testing was done. At time of enrolling in the current study, she had not spoken in 12 years, since her last surgery.

### Materials

Tests of receptive processing of phonemes included:

(1) *Word Discrimination* (Rogalsky et al., 2011)—The patient is auditorily presented with two single-syllable words and instructed to judge whether they are the same or different. Stimuli differed by only a single phonetic feature in the initial phoneme (i.e., minimal pairs, such as *Might-Night* or *Face-Vase*) and were matched for phonotactic frequency and phonological neighborhood density. There were 20 “same” trials and 20 “different” trials.

(2) *Nonword Discrimination* (Rogalsky et al., 2011)—The patient is auditorily presented with two single-syllable pseudowords and instructed to judge whether they are the same or different. As with the word discrimination task, stimuli differed by only a single phonetic feature in the initial phoneme, involved all of the same contrasts as in the word discrimination task, and were matched for phonotactic frequency and phonological neighborhood density. There were 20 same trials and 20 different trials.

(3) *Word-to-Picture Matching Phonological Foils* (Rogalsky et al., 2011)—The patient is auditorily presented with a probe word and instructed to choose which picture matches the word from among four alternatives. The alternatives are all phonological distractors that rhyme with the target. There were 20 total trials.

(4) *Adaptive Word-to-Picture Matching* (Stokes et al., 2019)—The patient was auditorily presented with a synthetic one-syllable word embedded in white noise followed by visual presentation of two black-and-white drawings on the left and right side of a computer screen, and the patient was instructed to press a key on the keyboard to indicate the matching picture. The volume of the auditorily presented word was adaptive, decreasing after correct trials and increasing after incorrect trials, while the volume of the white noise was constant. There were 200 trials per condition. There were four stimulus pair conditions (*pie-buy*, *tie-die*, *buy-die*, *pie-tie*). The order of auditory tokens from a given pair and the orientation of the pictures on the screen were randomized. A logistic psychometric curve was fit to the data, and the signal-to-noise ratio (dB) that yielded 75% performance accuracy is reported. Because dB is on a log scale, a positive value indicates that the signal was louder than the noise, a negative value indicates that the noise was louder than the signal, and zero indicates equal amplitudes for signal and noise. Lower dB values indicate better performance (greater noise tolerance); the maximum dB value was capped at 30 dB, indicating total failure to discriminate the target word, even with relatively negligible background noise.

(5) *Audiovisual Integration* (Hickok et al., 2018)—In the auditory-only condition, the patient was auditorily presented with different instances of a naturally spoken syllable by a male speaker from the set {/pa/, /ka/} and instructed to point to the written syllable that matched among three alternatives on a computer screen {/pa/, /ka/, /ta/}. There were 30 trials in this condition. In the audiovisual condition, the patient was auditorily presented with a syllable from the set {/pa/, /ka/} synchronously with a video of the same male speaker that was congruent or incongruent (auditory /pa/ with visual /ka/), and the patient was instructed to point to the written syllable that matched the auditory percept among three alternatives on a computer screen {/pa/, /ka/, /ta/}. Incongruent stimuli were pilot tested to include instances that were most likely to evoke a fused percept. There were 10 congruent /pa/ trials, 10 congruent /ka/ trials, and 10 incongruent trials.

Tests of receptive processing of words included:

(1) *Word-to-Picture Matching Mixed Foils* (Rogalsky et al., 2011)—The patient is auditorily presented with a probe word and instructed to choose which picture matches the word from among four alternatives. The alternatives include a semantic distractor from the same taxonomic category as the target, a phonological distractor that rhymes with the target, and an unrelated distractor. There were 20 total trials.

(2) *Word-to-Picture Matching Mixed Foils in Noise* (Rogalsky et al., 2011)—The patient is auditorily presented with a probe word embedded in 14 dB Gaussian white noise and instructed to choose which picture matches the word from among four alternatives. The alternatives include a semantic distractor from the same taxonomic category as the target, a phonological distractor that rhymes with the target, and an unrelated distractor. There were 20 total trials.

(3) *Word-to-Picture Matching Semantic Foils* (Rogalsky et al., 2011)—The patient is auditorily presented with a probe word and instructed to choose which picture matches the word from among four alternatives. The alternatives are all semantic distractors from the same taxonomic category as the target. There were 20 total trials.

(4) *The Western Aphasia Battery* (WAB; Kertesz, 1982), *Auditory Word Recognition*—The patient is instructed to identify items from a given category by pointing. Categories include real objects, drawn objects, shapes, letters, numbers, colors, furniture, body parts, fingers, right-left body parts. There were 60 total items.

Tests of receptive processing of sentences included:

(1) *WAB*, *Yes/No Questions*—Questions are presented verbally to the patient with the answer being either yes or no. There were 20 total questions.

(2) *WAB, Sequential Commands*—The patient is verbally presented with a command that requires a sequence of two to four actions. There were 8 total commands involving 19 actions.

(3) *Sentence-to-Picture Matching* (subject-relative, object-relative, active, and passive [SOAP]; Love & Oster, 2002)—A sentence is verbally presented to the patient, and the patient is instructed to choose which picture matches the sentence among three alternatives. The “semantic distractor” alternative includes a different subject, object, and action from the target, while the “syntactic distractor” alternative reverses the roles of the subject and object. The sentences had different syntactic constructions, including active voice, passive voice, subject-relative clauses, and object-relative clauses. There were 10 sentences of each type.

(4) *Sentence-Picture Verification* (SOAP)—Each of the subject-relative and object-relative sentences from SOAP were presented verbally with either the correct picture or the syntactic distractor picture, and the patient was instructed to judge whether the sentence matched the picture.

(5) *Sentence Plausibility* (Rogalsky et al., 2018)—The patient is auditorily presented with a sentence and instructed to judge whether it is semantically plausible. The sentences had different syntactic constructions, including active voice, passive voice, subject-relative clauses, and object-relative clauses. There were 20 sentences of each type.

Tests of short-term memory included:

(1) *Digit Span (Forward)*—The patient was auditorily presented with a sequence of non-repeating digits and instructed to point to the same sequence on a number line. The list length, beginning with just a single digit, increased until errors occurred on all trials. The number line was not visible during presentation of the sequence. There were 4 trials per list length.

(2) *Word Span*—The patient was auditorily presented with a sequence of non-repeating, one-syllable words, selected from the set {bags, cage, dump, gash, king, mock, peach, rake, shoe, tent}, and instructed to point to the same sequence on an alphabetically arranged response sheet. The response sheet was not visible during presentation of the sequence. The list length, beginning with just a single word, increased until errors occurred on all trials of a list length condition. There were 4 trials per list length condition.

(3) *Nonword Span*—The patient was auditorily presented with a sequence of non-repeating, one-syllable pseudowords, selected from the set {bav, coaf, dook, fave, giz, kag, mide, nabe, perb, roash, tast}, and instructed to point to the same sequence on an alphabetically arranged response sheet. The response sheet was not visible during presentation of the sequence. The list length, beginning with just a single pseudoword, increased until errors occurred on all trials of a list length condition. There were 4 trials per list length condition.

(4) *4-Digit Recall*—The patient was presented with a sequence of four digits and instructed to point to the sequence on a number line after a 15 second delay. The number line was not visible during presentation of the sequence or during the maintenance interval. There were 4 total trials.

Auxiliary tests included:

(1) *WAB, Picture Description*—The patient is presented with a picture of a picnic scene and instructed to describe what they see. Responses were written.

(2) *WAB, Object Naming*—The patient is presented with a set of real objects and instructed to name them. Responses were written due to mutism. There were 15 total objects. The safety pin, eraser, padlock, pipe, and matches were not presented. The gun was replaced by a mirror, and the pencil was replaced by a pen.

(3) *WAB, Reading Comprehension of Sentences*—The patient is presented with a written sentence (or pair of sentences) with the final word elided, and the patient is instructed to choose the final word from a set of four alternatives. There were 8 total sentences.

(4) *WAB, Writing to Dictation*—The patient is verbally presented with the sentence, “Pack my box with five dozen jugs of liquid veneer.” The patient is instructed to write the sentence.

(5) *WAB, Apraxia*—The patient is instructed to pantomime actions with the upper limb, face, imagined instruments, and imagined complex situations such as driving a car. There were 20 total actions.

### Testing Schedule

Participant 1 completed three testing sessions with six weeks intervening between each visit. The first testing session lasted approximately 3 h. During the first testing session, she completed the SOAP, Word-to-Picture Matching with Mixed Foils, Word Discrimination, Nonword Discrimination, Digit Span, Word Span, Nonword Span, WAB (written picture description, yes/no questions, auditory word recognition, sequential commands, object naming, reading comprehension of sentences, writing to dictation, and apraxia subtests), and three conditions of the Adaptive Word-to-Picture Matching task. Structural MRI (1.5 T) was also acquired during the first visit; 3 T and functional imaging were contraindicated due to an implanted medical device. The second testing session lasted approximately one and half hours. During the second testing session, she completed the object- and subject-relative conditions of Sentence-to-Picture Matching, Sentence Verification, Digit Span, 4-Digit Recall, Word Span, Nonword Span, Word-to-Picture Matching with Semantic Foils, Sentence Plausibility, and three conditions of the Adaptive Word-to-Picture Matching task. The three sentence processing tasks were added to the testing schedule to confirm and investigate the object-relative sentence processing deficit observed during the first testing session. The third testing session lasted approximately one hour. During the third testing session, she completed Word-to-Picture Matching with Phonological Foils, Word-to-Picture Matching with Mixed Foils in Noise, Audiovisual Integration, and four conditions of the Adaptive Word-to-Picture Matching task.

Participant 2 completed two testing sessions with eight weeks intervening. The first testing session lasted approximately 2 h. During the first testing session, she completed the SOAP, Word-to-Picture Matching with Mixed Foils, Word Discrimination, Nonword Discrimination, Digit Span, 4-Digit Recall, WAB (auditory verbal comprehension, word recognition, sequential commands, and apraxia subsections), and one condition of the Adaptive Word-to-Picture Matching task. Structural and functional MRI (3 T) was also acquired during the first visit. Functional MRI tasks included covert object naming, covert action naming, and covert famous face naming. During the second testing session, she completed Word Span, Nonword Span, Word-to-Picture Matching with Phonological Foils, Word-to-Picture Matching with Mixed Foils in Noise, Audiovisual Integration, and four conditions of the Adaptive Word-to-Picture Matching task.

### Neuroimaging

Anatomical MRI scans were obtained for Case 2 using a 3 T whole-body magnetic resonance scanner (Philips Medical Systems) fitted with a 16-channel SENSE head coil. Images were collected using a magnetization-prepared 180 radio-frequency pulse and rapid gradient-echo sequence with 1 mm sagittal slices and an in-plane resolution of 0.938 x 0.938 mm. The same specifications were used for Case 1, with the exception of a 1.5 T field strength and a transmit/receive head coil. Images and renderings were generated with MRIcroGL (https://www.nitrc.org/projects/mricrogl/). Lesion segmentations were drawn on axial slices by a trained neuropsychologist (author G.W.) using MRIcroGL and checked for accuracy by a neurologist (author N.T.). Cortical atrophy and ventricle dilation were not identified as part of the lesion segmentation. The central sulcus on each patient’s scan was traced by hand to provide an anatomical reference (with some approximation required inside the lesions).

## RESULTS

**Figure 1.**
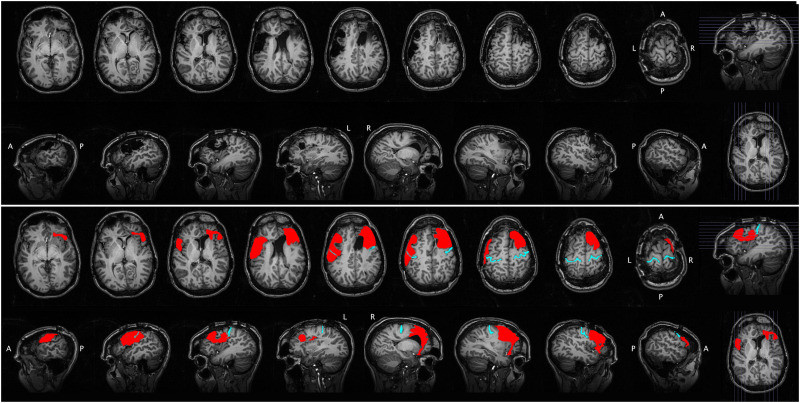
Structural MRI for Case 1. The raw scan is shown in the top portion of the figure, with axial slices (first row) and sagittal slices (second row). The same scan is shown in the bottom portion of the figure, with the lesion segmentation highlighted in red and the central sulcus highlighted in cyan for reference. A = anterior, P = posterior, L = left, R = right.

**Figure 2.**
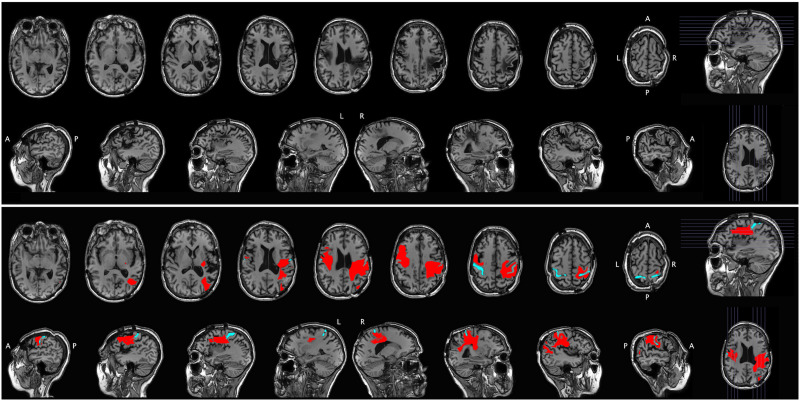
Structural MRI for Case 2. The raw scan is shown in the top portion of the figure, with axial slices (first row) and sagittal slices (second row). The same scan is shown in the bottom portion of the figure, with the lesion segmentation highlighted in red and the central sulcus highlighted in cyan for reference. A = anterior, P = posterior, L = left, R = right.

Results for all behavioral tests are presented in Table 1. Lesion maps are presented in Figure 1 and Figure 2.

**Table 1.** Behavioral test results for bilateral patients and neurotypical controls

**Test**	**Maximum score**	**Case 1**	**Case 2**	**Controls**
**Phonemes**				
Word discrimination (same)	20	18	20	
Word discrimination (different)	20	19	20	
Word discrimination A′	1	0.96	1	
Nonword discrimination (same)	20	16	20	
Nonword discrimination (different)	20	9	20	
Nonword discrimination A′	1	0.72	1	
Word-to-picture matching (phon foil)	20	19	20	[20, 20]
Adaptive word-to-picture matching (*buy-pie* 75% threshold)	-	21, >30 dB	>30 dB	[3.5, 8.5] dB
Adaptive word-to-picture matching (*die-tie* 75% threshold)	-	>30, 28, 19 dB	5.5, 1 dB	[−5.5, 2] dB
Adaptive word-to-picture matching (*buy-die* 75% threshold)	-	>30, 1.5, 19 dB	−14 dB	[−16, −14] dB
Adaptive word-to-picture matching (*pie-tie* 75% threshold)	-	>30, >30 dB	−2.5 dB	[−15, −8.5] dB
Audiovisual integration (A only)	30	29	30	
Audiovisual integration (A/V congruent)	20	19	20	
Audiovisual integration (A/V incongruent fuse/audio/visual)	10	3/5/2	10/0/0	
**Words**				
Word-to-picture matching (sem foil)	20	20	20	[20, 20]
Word-to-picture matching (mix foil)	20	20	20	[20, 20]
Word-to-picture matching (mix foil+noise)	20	16	17	[16, 19]
WAB word recognition	60	59	60	
**Sentences**				
WAB yes/no questions	20	20	20	
WAB sequential commands	19	15	19	
Sentence-to-picture matching (active)	10	9	10	
Sentence-to-picture matching (passive)	10	9	10	
Sentence-to-picture matching (subject relative)	10	9, 8	10	
Sentence-to-picture matching (object relative)	10	4, 3	9	
Sentence-picture verification, matching (subject relative)	5	5	-	
Sentence-picture verification, non-matching (subject relative)	5	4	-	
Sentence-picture verification, A′ (subject relative)	1	0.95	-	
Sentence-picture verification, matching (object relative)	5	4	-	
Sentence-picture verification, non-matching (object relative)	5	0	-	
Sentence-picture verification, A′ (object relative)	1	0	-	
Sentence plausibility, plausible (active)	10	6	-	
Sentence plausibility, implausible (active)	10	10	-	
Sentence plausibility, A′ (active)	1	0.90	-	
Sentence plausibility, plausible, (passive)	10	7	-	
Sentence plausibility, implausible (passive)	10	9	-	
Sentence plausibility, A′ (passive)	1	0.88	-	
Sentence plausibility, plausible, (subject relative)	10	7	-	
Sentence plausibility, implausible (subject relative)	10	9	-	
Sentence plausibility, A′ (subject relative)	1	0.88	-	
Sentence plausibility, plausible, (object relative)	10	7	-	
Sentence plausibility, implausible (object relative)	10	5	-	
Sentence plausibility, A′ (object relative)	1	0.67	-	
**Short-term memory**				
Digit span		4	5	6^a^
Word span		3	4	
Nonword span		2	3	
4-digit recall (15 s)	4	1	2	

*Note*. The range of control performance is reported in square brackets, [min., max.]. Multiple, non-bracketed, comma-separated entries within a cell reflect multiple attempts on different testing occasions. For the Adaptive Word-to-Picture Matching task, the signal-to-noise ratio (dB) that yielded 75% performance accuracy is reported. Because dB is on a log ratio scale, a positive value indicates that the signal was louder than the noise, a negative value indicates that the noise was louder than the signal, and zero indicates equal amplitudes for signal and noise. Lower dB values indicate better performance (greater noise tolerance); the maximum dB value was capped at 30 dB, indicating total failure to discriminate the target word, even with relatively negligible background noise. A′ represents “an estimate of proportion correct corrected for response bias” (see Rogalsky, Pitz, et al., 2008, p. 168). A: audio; A/V: audio-visual; phon: phonological distractor; sem: semantic distractor; mix: mixed distractor. A only: audio signal only; A/V congruent: audio-visual signals from the same production.

^a^ Selnes et al., 1991 – 5th percentile.

### Word and Nonword Discrimination

Both participants performed well on the word discrimination task: Case 1 scored 92.5% (A′ = 0.96) and Case 2 scored 100%. (A′ represents “an estimate of proportion correct corrected for response bias”; see Rogalsky, Pitz, et al., 2008, p. 168). The two cases diverged in their ability to perform the nonword discrimination task. Case 1 had significant difficulty, scoring only 62.5% (A′ = 0.76), whereas Case 2 scored 100%. This suggests that Case 1 has a verbal short-term memory deficit that limits her ability to maintain and compare two phonological forms without lexical-semantic support. In a follow-up analysis, we assessed whether we could induce a dissociation between word and nonword discrimination by testing two of the authors (neurotypical listeners) on a version of these tasks where the stimuli were presented in noise to bring performance down from ceiling levels. Performance was indeed worse on the noise version of the tasks but was similar for words (listener 1: 80% correct, listener 2: 85% correct) versus nonwords (listener 1: 80% correct, listener 2: 82.5% correct). Thus, the dissociation observed in Case 1 is likely not attributable to general differences in the difficulty of the two versions.

Performance on the adaptive word-comprehension in noise task was relatively poor for both patients. Case 1 was essentially at floor levels, converging on an identifiable threshold on only 2 out of 40 staircase runs (both in the easiest, *buy*/*die*, condition). Case 2 exhibited mixed performance on this task, performing poorly on two of the phonemic contrasts (*pie*/*tie* and *buy*/*pie*) and within normal limits on the other two contrasts (*die*/*tie* and *buy*/*die*).

### Audiovisual Tasks

Both participants performed well on the auditory only and congruent audiovisual (AV) task conditions, scoring 95% correct or better. For the incongruent AV task condition, Case 2 showed a robust McGurk effect, reporting fused percepts on every trial. Case 1’s phonemic perception was also influenced by incongruent AV stimuli, reporting a percept corresponding to the audio signal only 50% of the time (cf. 95% in the auditory only condition); the remaining 50% of her responses were fused (3/5) and visual (2/5).

### Word Comprehension

Both patients performed nearly flawlessly on all tests of non-noisy word comprehension (word-picture matching with phonological, semantic, and mixed foils, WAB Word Recognition). Case 1 scored 98.3% correct (118/120) and Case 2 scored 100% on these tests. Performance was within the normal range on the mixed foil word comprehension in noise task for both patients, although lower, of course, than performance on the clear speech version of the task. Thus, word comprehension appears well-preserved in both patients.

### Sentence Comprehension

Comprehension of simple sentences was largely preserved for both of our participants, who scored perfectly on the WAB yes/no questions and 90% or better on the sentence-picture matching task for active, passive, and subject-relative sentences. When the sentence structure increased working memory load (WAB sequential commands and object-relative sentences), Case 1’s performance dropped to 66% correct whereas Case 2 performed well (97% correct). Case 1 exhibited a strong primacy effect on the sequential commands task; all of the initial actions were correct. Given the marked deficit that Case 1 exhibited for object-relative processing on the original sentence comprehension task, follow-up tests were administered that reduced the working memory demands associated with choosing among response alternatives. Still, a similar working memory load pattern was observed for Case 1 on the sentence verification and plausibility tasks, with notable deficits for object-relative structure only, indicating that the deficit observed in sentence-picture matching did not depend on selection among multiple response alternatives. Case 1 exhibited a strong bias toward “yes” answers on the object-relative sentence verification task, suggesting a reliance on identification of lexical items instead of their syntactic roles.

### Span Tasks

Both participants showed some evidence of reduced short-term memory. Case 1 performed consistently worse than Case 2 across the tasks, which is consistent with her poorer performance on language tasks that involve higher short-term memory load.

### Additional Observations

Case 1 exhibited mild aphasic deficits on written production tasks. She scored 13/15 on WAB written naming (rubber band → “rubber bamb”, screwdriver → “flatliner”). Written picture description initially consisted only of single words (picnic, sailboat, beach, fishing, house, lunch). After being prompted to use complete sentences, she produced a few short phrases (“Man has his shoes off”; “Dog chasing a kid”; “It’s windy”; “It’s sunny also windy”; “Car has no color”). On writing to dictation of *Pack my box with five dozen jugs of liquid veneer*, she wrote, “Pack my box with 5 dozens of jugs of liquid vanery.” Although there was not enough time to formally evaluate written production for Case 2, her use of her smartphone for text-to-speech communication was rapid, fluent, and errorless. During a break from testing, she communicated about her memories of being in the hospital for surgery, her family medical history, and suggestions for sightseeing while visiting her hometown. Both participants used smartphones for text or text-to-speech communication in their daily lives.

Case 1 was completely unable to phonate voluntarily, although her mother reported that she could (very rarely) produce spontaneous vocalizations in response to pain or fear, such as when waking from a nightmare. Case 2 was able to voluntarily produce guttural sounds using the larynx with rising or falling pitch, which she used to communicate, for example, indicating confusion/understanding or affirmative/negative, often paired with appropriate head movements and facial expressions involving the muscles around the eyes and the brow. The spontaneous Duchenne smile was observed in both cases during testing. Both patients reported having the experience of inner speech.

Both patients were able to pantomime upper limb gestures (e.g., making a fist), instrumental gestures (e.g., using a hammer), and complex gestures (e.g., pretending to knock at the door and open it). Both patients were also able to close their eyes on command, and were able to attempt gestures with the lower face. Case 1 was able to pantomime sniffing a flower and blowing out a match, but was unable to stick out her tongue or whistle. Case 2 was unable to perform any of these lower face gestures, and was unable to attempt whistling. Case 1 was able to take food orally, including sucking on a breath mint and drinking from a cup or straw. Case 2 had dysphagia and so was unable to take food orally, relying on a feeding tube; she used a hand towel to manage involuntary production of saliva that could not be swallowed.

### Structural Neuroimaging

Case 1 has a large left fronto-parietal lesion centered on lower sensorimotor and premotor (BA6) cortex. The lesion extends anteriorly to include the pars opercularis and most if not all of the pars triangularis; inferior portions of the posterior middle frontal gyrus are also involved. Posteriorly, the lesion extends into the inferior parietal lobule but spares the posterior supramarginal gyrus. In the right hemisphere, the lesion is restricted to the frontal lobe, much of which is involved, including lateral premotor cortex, Broca’s area homolog, and inferior motor cortex. *Spared* regions include the frontal pole, ventromedial structures, posterior dorsomedial structures, mid-superior portions of the precentral gyrus, and portions of the frontal operculum.

Case 2 turned out to have more asymmetric damage. In the left hemisphere, damage is centered on the pars opercularis/premotor cortex with posterior extension into inferior primary sensorimotor cortex. The pars triangularis of Broca’s area is largely spared. In the right hemisphere the lesion is predominantly in the parietal lobe involving virtually all of it from the postcentral gyrus (including the portions of the lower precentral gyrus) to posterior parietal cortex (even including portions of the angular gyrus, but with some islands of sparing around the supramarginal gyrus) and from the parietal operculum to dorsal and medial parietal areas, sparing only the posterior cingulate and posterior precuneus region. The lesion extends in depth to the lateral ventricle, thus interrupting most of the dorsal stream white matter pathways.

## DISCUSSION

We evaluated the receptive speech and language processing abilities in two cases of opercular syndrome. Both have damage to left hemisphere motor speech areas including lower primary sensorimotor cortex, lower premotor cortex (BA6), and the pars opercularis of Broca’s area. Case 1 also has involvement of right hemisphere motor speech areas (M1, premotor cortex, Broca’s area), whereas Case 2 has an extensive parietal lobe lesion interrupting dorsal stream sensorimotor processing. Behaviorally, both were incapable of voluntary control of their vocal tract and lower facial muscles (although Case 2 was able to produce guttural sounds for limited communication), consistent with opercular syndrome. Despite these severe motor speech deficits, both participants performed remarkably well on the clear speech word-to-picture matching comprehension task and word-pair discrimination tasks, even when subtle phonemic cues had to be resolved for correct performance. Case 1 had some difficulty with nonword discrimination and with the identification of synthesized speech in noise, which we argue stems from a phonological short-term/working memory deficit (see below). Both participants showed sensitivity to mismatched AV speech signals and were able to comprehend simple sentences. Case 2 was also able to comprehend syntactically complex sentences showing largely preserved receptive language abilities despite her motor speech impairment. Case 1 had difficulty with complex syntax and limited immediate recall span, consistent with a phonological short-term memory (pSTM) deficit. A previous report in the literature identified a case of opercular syndrome with similar difficulty comprehending center embedded sentences, which they also linked to a pSTM deficit (Silveri et al., 2017).

### Implications for Motor-Based Theories of Speech Perception

The present study adds to the large body of evidence arguing against a strong version of the motor theory of speech perception, that an intact motor speech system is required to perceive speech. Both of the present cases performed well on the word-to-picture matching tests and the word discrimination test, even though the ability to distinguish between highly similar phones was required for success. A strong version of the motor theory predicts impaired performance on all tasks requiring this degree of phonemic discrimination.

Weaker, modulatory theories of the role of the motor speech system in perception can claim some tentative, qualified support, however. Specifically, Case 1, who had more extensive bilateral motor involvement, was impaired on nonword discrimination and was at floor on our adaptive speech-in-noise task. If one assumes that Case 2, whose right precentral motor system was largely spared, performed well on these same tasks *because* of the sparing of her right motor system, then one could argue that the motor system contributes substantially to the performance on these tasks. This is a *tentative* conclusion because Case 2 became anarthric only after the right hemisphere event, indicating that the right hemisphere lesion, in combination with the left hemisphere lesion, indeed permanently interrupted motor speech function. Nonetheless, the discussion below will assume, for the sake of argument in favor of motor theories, that the difference in performance between Case 1 and Case 2 is due to the difference in motor cortex involvement.

The support for a weak version of the motor theory is *qualified* because our data suggest that it is task-dependent, holding only for tasks that involve nonwords or degraded stimuli presented in noise. The contrast in performance between the word and nonword discrimination tasks is particularly telling. For both tasks, listeners have to make fine phonemic discriminations in order to detect the difference between the items in each pair. Case 1 could perform this task successfully when the items were real words, but largely failed when the items were nonwords. Success on the word stimuli indicate that fine phonemic discrimination is well preserved and enables access to lexical-semantic representations. Given this, failure on the nonword discrimination task cannot be a result of phonemic perception impairment generally—otherwise word discrimination would be impossible—but rather, it depends on the ability to make a discrimination on the basis of phonemic information alone, since lexical-semantic access is not possible for nonwords. This suggests a pSTM explanation of Case 1’s difficulty with the nonword task, as we detail later.

Case 1 also had much more trouble on the adaptive word-comprehension-in-noise task than Case 2 had, performing essentially at floor. Taken at face value, this could be interpreted as support for the claim that the motor system is important for speech perception under noisy listening conditions. However, Case 1’s performance was much better (80% correct) and well above chance (25%) on the speech-in-noise word-picture matching task with natural speech stimuli. Indeed, this level of performance is just a single error below the performance range of controls tested as part of another study and in an acoustically controlled setting (91% correct, *SD* = 4.9%, range 85–100%; Rogalsky et al., 2011, unpublished data). It is also *within* the range of scores, albeit on the low end, from the four control participants tested in the same room under the same conditions and similar to Case 2’s performance (85% correct). If the dramatic failure on the adaptive task was a result of the lack of motor system input to perceiving speech in noise, we would expect a more substantial deficit on our other speech-in-noise task. This suggests that the adaptive task is tapping into something different. We will argue that it is again attributable to a short-term phonological memory problem. We turn to this issue next.

### Short-Term Memory, Speech Perception, and the Motor System

It is well-established that the motor speech system plays a critical role in pSTM via the articulatory rehearsal component of the phonological loop (Baddeley, 1992; Buchsbaum et al., 2011; Buchsbaum & D’Esposito, 2008; Hickok et al., 2003). Severe damage to the motor speech system, as in our present cases, should, therefore, cause pSTM deficits, which we observed, particularly for Case 1. The question we address here is whether a pSTM deficit can confound performance on speech perception tasks. That is, do some speech perception tasks rely in part on pSTM?

Case 1 performed poorly on our nonword discrimination task. This clearly involves some kind of pSTM, because the first item must be maintained until after the second item is presented, and then the two representations are compared. Because the items are nonwords, there is no opportunity to recode the stimuli semantically. This places the burden on some form of either auditory or phonological STM. Thus, there is no question that discrimination tasks require some degree of STM that is not required during word comprehension, which involves processing a single word-form and activating its associated lexical representation incrementally even before the whole word is perceived (W. D. Marslen-Wilson, 1987; W. Marslen-Wilson & Tyler, 1980). But, one might argue, listening to only two items would seem to impose a minimal pSTM burden, and even Case 1 had a nonword span of two items. However, the items in the nonword span task were phonologically dissimilar, which benefits pSTM (Baddeley, 1992), in contrast to the minimal pair comparisons in the discrimination task. Thus, the nonword span task may overestimate the STM resources available for performance on the discrimination task.

Case 1 also performed very poorly on our adaptive word-to-picture matching task, which involved degraded (synthesized) speech stimuli presented in noise. On this task, participants were not instructed to perform discrimination directly (i.e., they were instructed to match a word to a picture, rather than compare two word-forms), and so it should not tax pSTM in the same way as the discrimination task. As such, Case 1’s poor performance might be viewed as support for the motor system’s involvement in augmenting speech perception under noisy listening conditions (Moineau et al., 2005; Wilson, 2009), perhaps using some form of predictive coding (Hickok et al., 2011; Londei et al., 2009; Schwartz et al., 2012). On closer inspection, however, we noticed that some of the synthesized tokens were difficult to identify when presented in isolation (informal testing confirmed this; stimuli publicly available at https://osf.io/pw35n/) and were only identifiable when the target words were cued by pictures. We further noted two additional complicating aspects of the task. First, even with the pictures cueing the word category alternatives, the perceived ease of categorization of the synthesized speech stimuli was enhanced with more exposure to the two competitors as the staircase run proceeded during the relatively favorable signal-to-noise ratio trials; this effect was most noticeable on the most ambiguous stimuli (*pie*/*buy*). In other words, some degree of perceptual differencing between time-separated items in the stimulus set was important for optimal task performance. This differencing could tax pSTM. Second, the position of the two alternative pictures (and therefore response buttons) was randomized: On some trials alternative A appeared on the left with B on the right, and on other trials, it was reversed. This randomization places additional short-term and working memory demands on the participant, who must not only categorize the stimulus, but also match the decision to the correct response button, which varies from trial to trial. In short, the adaptive task is nontrivial. In this context, it is worth pointing out that in the prior study of 24 healthy undergraduates (Stokes et al., 2019), for which the task was designed, 4 (16.7%) were excluded due to failure to converge on two or more staircase runs, indicating that the task is indeed quite difficult for a sizeable fraction of even healthy listeners.

These observations suggest several explanations of the present speech-in-noise effect that are alternatives to a motor-prediction or analysis-by-synthesis mechanism (Bever & Poeppel, 2010). The first is that Case 1 may have fallen into the group of people who have difficulty with this task even prior to her surgery. That is, based on available data (Stokes et al., 2019), there is a 16.7% chance that she would have had a premorbid difficulty with the task that has nothing to do with her current lesions. A second possible explanation is that her frontal lobe lesions impaired not her ability to hear speech in noise, but her cognitive control ability (Brownsett et al., 2014; Gläscher et al., 2012; Novick et al., 2005), which could interfere with the performance of changing perception-response mappings. The third possible explanation is that the degraded nature of the synthesized speech stimuli themselves, even without noise, makes the task closer to a nonword discrimination task than a speech-in-noise task. The logic here is that because the auditory stimuli on their own map poorly onto word categories, the listener must first discriminate the two (nonword) alternatives and then map them onto word categories rather than directly comprehend each stimulus in an ecologically natural way. This suggestion is consistent with the well-known fact that the range of stimulus exemplars and comparison categories in the stimulus set influences category judgements for speech (Holt & Lotto, 2010) and implies some form of short-term memory of the stimulus set for task optimization. Thus, Case 1’s apparent pSTM deficit, as revealed by her impaired performance on immediate serial recall, sequential commands, object-relative sentence comprehension, and nonword discrimination tasks, is consistent with this analysis of her failure on the adaptive task. While Case 2 did not exhibit a particularly large advantage on immediate serial recall tasks over Case 1, the advantage was consistent across stimulus conditions; the different STM ability levels between these patients possibly straddled a critical threshold required for supporting speech processing in other tasks.

We conclude that motor speech deficits impact pSTM as both of our cases had reduced spans. If severely impaired, pSTM can impact performance on some speech perception tasks.

### Sentence Comprehension

Both of the present cases showed well-preserved comprehension for simple sentences, showing that receptive speech ability up to the level of basic sentences is not impeded by severe motor speech dysfunction. Case 1, however, had notable difficulty with syntactically complex sentences, such as semantically reversible object-relative structures. She also had difficulty with multistep sequential commands. Comprehension of such complex sentences is known to activate frontal, motor-related language areas (for reviews see Friederici, 2003; Matchin & Hickok, 2020; Rogalsky, Matchin, & Hickok, 2008; Segaert et al., 2012). Some have argued that regions such as Broca’s area play a key role in syntax, including during comprehension (Fedorenko & Kanwisher, 2011; Friederici, 2003; Segaert et al., 2012), while others argue that frontal areas are only involved when working memory demands are high (Matchin & Hickok, 2020; Rogalsky, Matchin, & Hickok, 2008). The present findings do not strongly disentangle these claims: It could be that Case 1’s pSTM deficit is the cause of her sentence comprehension problems, or she might have both a pSTM deficit and a separate syntactic deficit due to her frontal lesion. Case 2, with her relatively preserved complex sentence comprehension, might have adjudicated, but her lesion appears to spare at least the pars triangularis of Broca’s area, leaving open the possibility that this region is still available to support syntactic ability.

### Audiovisual Speech

Dominant neural models of audiovisual speech integration hold that the posterior superior temporal sulcus is a critical site (Beauchamp et al., 2004; Beauchamp et al., 2010; Venezia et al., 2017). Other models implicate motor speech networks (Sams et al., 2005; Skipper et al., 2005, 2007). The present cases enabled a test of the role of motor speech systems in AV speech integration by assessing whether a McGurk effect would emerge when audio and visual cues were in conflict. Both cases showed sensitivity to AV mismatched stimuli, in that they deviated from their performance on audio-only perception. Case 2 showed a robust McGurk effect, reporting a fused percept on every trial. This demonstrates that the intact motor control of speech is not necessary for McGurk fusion. A similar conclusion comes from a large-scale study of unilateral lesions, which found that posterior, not anterior damage predicted AV integration failures (Hickok et al., 2018). Case 1 reported fused syllables on 30% of the trials. The interpretation of this pattern is unclear because not all healthy individuals exhibit McGurk fusion (Basu Mallick et al., 2015).

### Conclusions

The main finding from this study is that bilateral damage to the motor speech system has little effect on the ability to recognize speech even when motor speech output is extremely impaired Such damage can impair pSTM span and, in Case 1, produce an agrammatic-type comprehension pattern. Audiovisual speech integration is not necessarily impaired, consistent with a recent large-scale lesion study, nor is speech-in-noise perception necessarily profoundly affected. Typical of neuropsychological case reports, the neurological interventions studied here were undertaken for medical, not scientific, purposes, and, as such, the motor speech system was not affected exclusively or completely. Furthermore, the continued presence of epileptic disorder, at least in one participant, and the considerable length of time between the surgical interventions and our assessments, allow for the possibility of reorganization of functional brain networks. It is noteworthy, however, that despite all of the opportunity for reorganization of motor speech systems, this was not found for expressive function. The possibility remains open that the motor system does play a nontrivial role in receptive speech processing under typical conditions, but that when it is destroyed, sensory systems alone can ultimately achieve the same or very nearly the same level of performance. But this is fundamentally what we are arguing: The motor system is not critical for speech perception. These findings confirm that a strong version of the motor theory of speech perception is untenable and provide only weak support for weaker versions of the motor theory, in which the motor system plays a small role at best in perceiving speech under near threshold conditions for only some types of speech stimuli.

## FUNDING INFORMATION

This research was supported by funding from the National Institute for Deafness and Other Communication Disorders P50 DC014664-5340 (Hickok), the National Institute of Neurological Disorders and Stroke R01 DC014589 (Tandon), and UT Health start-up for Texas Institute of Restorative Neurotechnologies (Tandon).

## AUTHOR CONTRIBUTIONS

**Grant M. Walker**: Data curation: Equal; Formal analysis: Equal; Investigation: Lead; Methodology: Equal; Project administration: Supporting; Visualization: Lead; Writing – original draft: Lead; Writing – review & editing: Equal. **Patrick Sarahan Rollo**: Data curation: Equal; Investigation: Supporting; Writing – review & editing: Supporting. **Nitin Tandon**: Conceptualization: Equal; Formal analysis: Equal; Funding acquisition: Lead; Methodology: Equal; Project administration: Supporting; Resources: Lead; Supervision: Lead; Visualization: Supporting; Writing – review & editing: Equal. **Gregory Hickok**: Conceptualization: Lead; Funding acquisition: Supporting; Methodology: Equal; Writing – original draft: Supporting; Writing – review & editing: Equal.

## TECHNICAL TERMS

Sublexical: Refers to language representations that are smaller than a word, such as syllables or individual speech sounds.
Anarthria: The complete inability to control the muscles for articulating speech.
Wada procedures: Involve injecting anesthetic into one hemisphere of the brain to deactivate it.
Operculum: A region on the lateral surface of the brain that includes portions of primary motor and sensory areas.
Apraxia: A disorder of motor planning for actions.
MRIcroGL: Free software for visualizing and editing neuroimaging data.
Axial: The plane passing through the head that gives a top-down or bottom-up view; both hemispheres are visible.
Central sulcus: An indentation or “valley” on the brain’s surface, between primary motor and sensory areas.
The McGurk effect: When incongruent auditory and visual speech signals are perceived as a completely different speech sound.
